# Advances in virus-like particle-based SARS-CoV-2 vaccines

**DOI:** 10.3389/fcimb.2024.1406091

**Published:** 2024-06-26

**Authors:** Xiaoting Hao, Feifei Yuan, Xuan Yao

**Affiliations:** ^1^ Department of Teaching Administration, Xiangyang No.1 People’s Hospital, Hubei University of Medicine, Xiangyang, China; ^2^ Department of Reproductive Medicine, Xiangyang No.1 People’s Hospital, Hubei University of Medicine, Xiangyang, China; ^3^ Department of Neurology, Xiangyang No.1 People’s Hospital, Hubei University of Medicine, Xiangyang, China

**Keywords:** COVID-19, SARS-CoV-2, vaccines, spike protein, receptor binding domain, virus-like particles, immune response

## Abstract

The Coronavirus Disease 2019 (COVID-19) pandemic, caused by the severe acute respiratory syndrome coronavirus 2 (SARS-CoV-2), has incurred devastating human and economic losses. Vaccination remains the most effective approach for controlling the COVID-19 pandemic. Nonetheless, the sustained evolution of SARS-CoV-2 variants has provoked concerns among the scientific community regarding the development of next-generation COVID-19 vaccines. Among these, given their safety, immunogenicity, and flexibility to display varied and native epitopes, virus-like particle (VLP)-based vaccines represent one of the most promising next-generation vaccines. In this review, we summarize the advantages and characteristics of VLP platforms, strategies for antigen display, and current clinical trial progress of SARS-CoV-2 vaccines based on VLP platforms. Importantly, the experience and lessons learned from the development of SARS-CoV-2 VLP vaccines provide insights into the development of strategies based on VLP vaccines to prevent future coronavirus pandemics and other epidemics.

## 1 Introduction

Coronavirus Disease 2019 (COVID-19) is an emerging disease caused by the novel human coronavirus (CoV), severe acute respiratory syndrome coronavirus-2 (SARS-CoV-2). Since its initial outbreak in December 2019, COVID-19 has posed a global public health threat (Ciotti et al., 2020; Zhou et al., 2020; Hu et al., 2021), with more than 774 million infections and over 7.0 million deaths as of February 11, 2024 (https://covid19.who.int/, accessed on February 11, 2024). In contrast to the two other prominent zoonotic beta-coronaviruses, SARS-CoV and Middle East respiratory syndrome coronavirus (MERS-CoV), SARS-CoV-2 has undergone continuous mutations, leading to the emergence of multiple variants of concern (VOCs), including the Alpha, Beta, Gamma, Delta, and Omicron variants (Kirtipal et al., 2020). The latest variant, Omicron, has mutated into subvariants including BA1, BA2, BA3, BA4, and BA5, with the dominant subvariants including EG.5, XBB.1.5, BA.2.86, XBB.1.16., BQ.1.1, and JN.1 (Parsons and Acharya, 2023; Yang et al., 2024). These newly emerged Omicron subvariants are rapidly transmitted among humans and are resistant to current vaccines and therapeutic antibodies that target the wild-type strain and previous VOCs (Cox et al., 2023; Wang et al., 2023; Zou et al., 2023; Li et al., 2023a). The evolution of these Omicron subvariants has lengthened the fallout of the COVID-19 pandemic, which exerts significant social and financial impacts worldwide. Vaccines are the most effective and economical means of controlling SARS-CoV-2 and its variants. Therefore, an urgent need is to develop an improved vaccine against the current SARS-CoV-2 variants to prevent future pandemics.

In this review, we summarize the structure and functions of the spike (S) protein and its receptor-binding domain (RBD) of SARS-CoV-2, which are critical targets of COVID-19 vaccines, and provide an overview of virus-like particle (VLP) vaccines against SARS-CoV-2 in the pre-clinical and clinical stages. In addition, we discuss the potential challenges that may hinder the development of VLP vaccines and propose potential strategies to prevent future coronavirus pandemics.

## 2 Structure and functions of SARS-Cov-2 S protein and RBD

**Figure 1:**
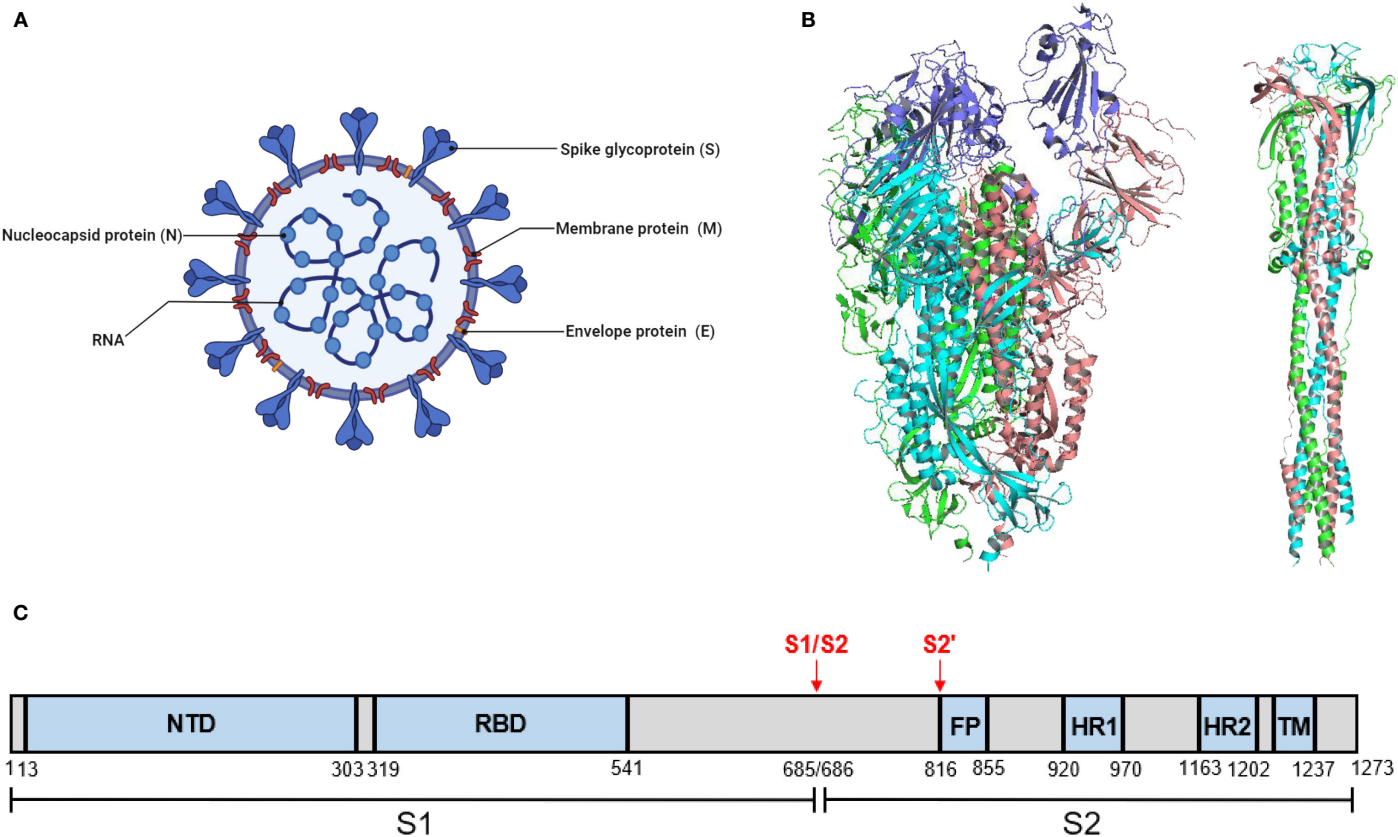
SARS-CoV-2 virion and the structure of its S protein. **(A)** A schematic diagram of SARS-CoV-2 virion. This figure was adapted from BioRender.com. **(B)** Structure of SARS-CoV-2 S protein. (left) The prefusion structure of the S protein with one RBD in the “up” conformation (PDB: 6VSB). (right) The post-fusion structure of the S protein (PDB: 6M3W). **(C)** Schematic domain structure of SARS-CoV-2 S protein. The S1 and S2 subunits are indicated, with the locations of furin cleavage sites. SP, signal peptide. NTD, N-terminal domain. RBD, receptor-binding domain. FP, fusion peptide. HR1 and HR2, heptad repeat 1 and 2. TM, transmembrane.

SARS-CoV-2 comprises four main structural proteins, including the spike (S), envelope (E), membrane (M), and nucleocapsid (N) proteins (**Figure 1A**). The S protein of SARS-CoV-2 exists as a homotrimer, which has been reported in both pre-fusion and post-fusion forms (**Figure 1B**). The S protein is composed of two functional subunits, S1 and S2. The S1 subunit comprises an N-terminal domain (NTD) and a receptor-binding domain (RBD) responsible for receptor binding. The S2 subunit comprises four structural regions: the fusion peptide (FP), two heptad repeats (HR1 and HR2), and a transmembrane domain (TM) that mediates membrane fusion (**Figure 1C**). Fusion of SARS-CoV-2 with host cells is critically dependent on the furin cleavage site between the S1 and S2 subunits. In host cells, the S protein is proteolytically cleaved at the PRRAR motif (residues 681–685) by furin into S1 and S2 to form an S1/S2 heterodimer, which is further assembled into a final trimeric spike complex. In addition, the S2 subunit contains an S’ cleavage site, cleaved by host proteases like transmembrane protease serine 2 (TMPRSS2) to expose the fusion peptide for membrane fusion (Jackson et al., 2022; Takeda, 2022). In the prefusion state, the homotrimeric S protein primarily adopts two conformations, a closed form with all RBD domains in the “down” conformation and an open form with one or multiple RBDs in the “up” conformation. Notably, only the RBD with an ‘up’ conformation can bind to the host receptor angiotensin-converting enzyme 2 (ACE2) (Yan et al., 2021). Moreover, the ubiquitous expression pattern of ACE2 receptors in various human organs, including the lungs, heart, brain, and kidneys, explains the tropism of SARS-CoV-2.

S protein plays a critical role in SARS-CoV-2 infection and pathogenesis. Critically, the S protein induces specific neutralizing antibodies (nAbs) that can block the RBD-human angiotensin-converting enzyme 2 (hACE2) interaction on host cells to prevent infection. Although all structural proteins of SARS-CoV-2 can induce nAbs, the S protein is highly immunogenic, and the RBD of the S protein is the most immunodominant target for inducing nAbs (Guan et al., 2023). Therefore, the S protein and its RBD constitute two major antigen targets for the development of SARS-CoV-2 vaccines.

## 3 Current updates on vaccines against SARS-CoV-2

**Figure 2:**
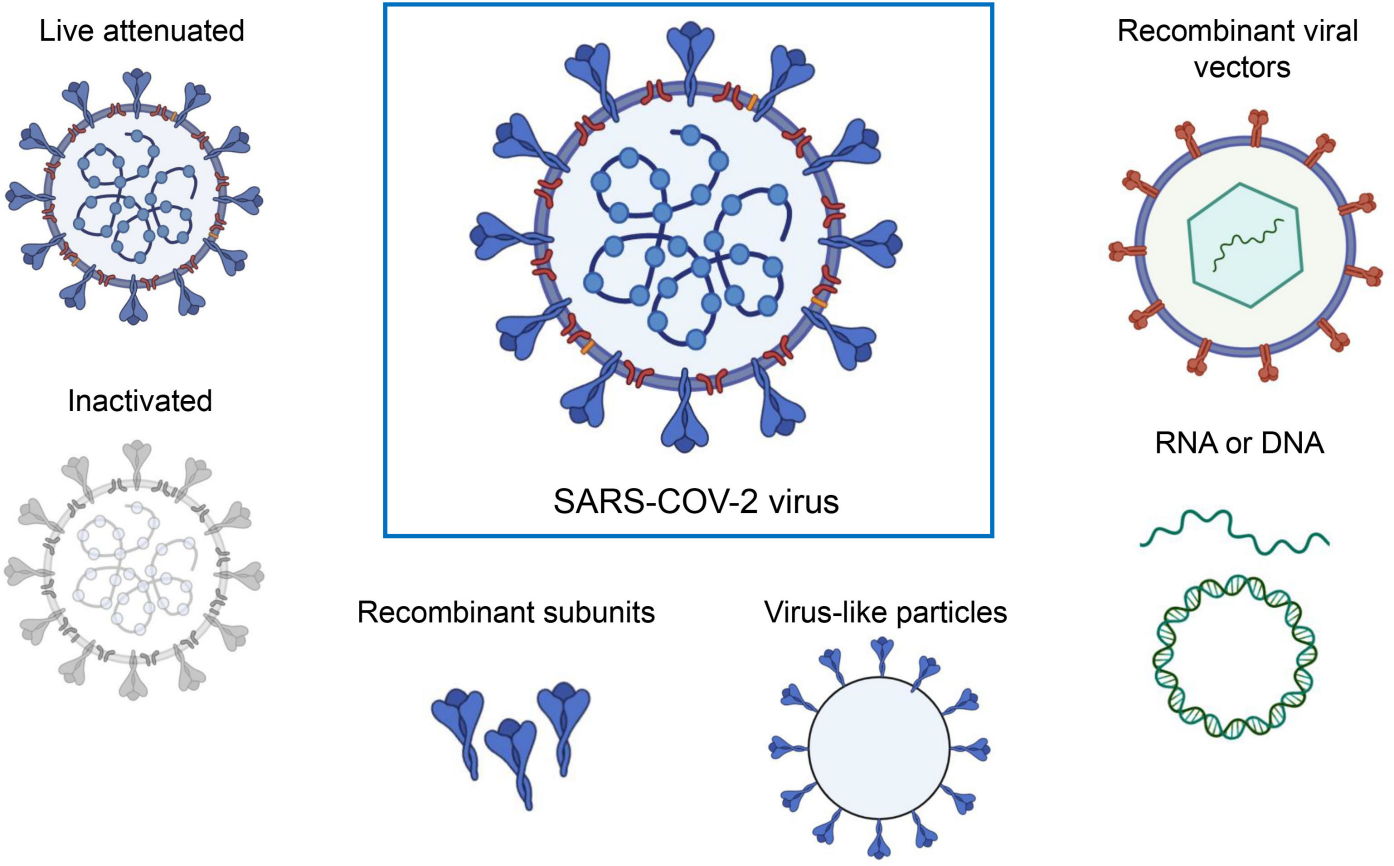
A range of different technologies for developing SARS-COV-2 vaccines. The figure was adapted from BioRender.com.

Currently, various platforms are devoted to producing SARS-CoV-2 vaccines, including mRNA, viral vectors, virus-like particles, protein subunits, and inactivated and live attenuated viruses. (**Figure 2**). According to the World Health Organization (WHO) database, as of March 30, 2023, the global SARS-CoV-2 vaccine research and development (R&D) landscape included 382 candidate vaccines, of which 183 are in clinical development and 199 were in the pre-clinical stage. Among the 183 candidate vaccines in clinical development, protein subunit vaccines are the most abundant, at 59, accounting for 32%, followed by RNA vaccines (24%), viral vector (non-replicative) vaccines (14%), inactivated vaccines (12%), and DNA vaccines (9%). Virus-like particle (VLP) vaccines account for 4% of all vaccines (**Table 1**) (https://www.who.int/publications/m/item/draft-landscape-of-covid-19-candidate-vaccines, accessed on March 30, 2023).

**Table 1:** Technical type of SARS-COV-2 vaccine candidates in clinical studies.

Platform of vaccine candidates	Numbers (%)
Protein subunit	59 (32%)
RNA	43 (24%)
Viral Vector (non-replicating)	25 (14%)
Inactivated Virus	22 (12%)
DNA	17 (9%)
Virus Like Particle	7 (4%)
Others	10 (5%)
	183 (100%)

## 4 VLP-based SARS-CoV-2 vaccines

A virus-like particle vaccine is a highly structured protein nanoparticle assembled from single or multiple structural proteins, which are usually similar in morphology to natural viral particles. Therefore, it is known as a virus-like particle vaccine in the academic community or simply a VLP vaccine. The first VLP-based vaccine licensed for humans in 1981 was the recombinant hepatitis B virus (HBV) vaccine, which comprises self-assembling particles formed by the recombinant expression of the hepatitis B virus (HBV) surface antigen (HBsAg) with a size of 22 nm (Krugman, 1982). Notably, HBsAg particles are devoid of nucleic acid, and are therefore non-infectious (Pumpens and Grens, 2016). Since then, the success of the HBV VLP vaccine has driven the development of investigational conjugate vaccines, wherein antigens from other pathogens are loaded onto the HBV platform. RTS,S/AS01, developed by GSK, is a VLP vaccine against malaria based on a portion of the major circumsporozoite protein of *Plasmodium falciparum* (PfCSP) fused to HBsAg. The European Union approved this vaccine with acceptable safety, efficacy, and tolerability profiles (RTS,S Clinical Trials Partnership, 2015). The World Health Organization recommends widespread vaccination of children with this vaccine in sub-Saharan Africa and other areas with moderate to high transmission of *Plasmodium falciparum*. Recently, another malaria vaccine, R21/Matrix-M, based on HBsAg particles, has also been approved by WHO for use in children in Africa (Datoo et al., 2024). In 2011, a VLP vaccine against hepatitis E was licensed for human use in China (Zhu et al., 2010). Other VLP-based vaccines targeting human papillomavirus (HPV) infections have also been approved for human use worldwide (Harper and DeMars, 2017). The HPV L1 major capsid protein self-assembles into hollow 55 nm VLPs displaying 72 pentameric antigens. The lack of immunogenicity after vaccination with denatured L1 protein subunits illustrates the requirement for the correct assembly and display of antigenic epitopes (Breitburd et al., 1995).

**Figure 3:**
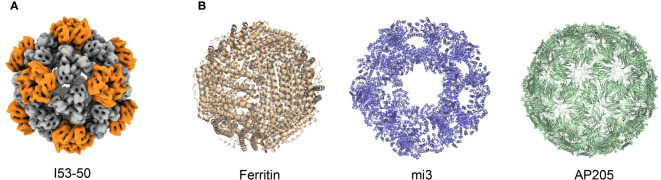
Structure of different VLPs. **(A)** Structure of I53–50 from EMDataResource (EMD-0350). **(B)** Structures from Protein Data Bank (PDB), PDB codes are the following: 3EGM (Ferritin), 7B3Y (mi3), and 5LQP (AP205).

In addition to HBV and HPV VLP vaccines, which self-assemble nanoscale particles using antigens on the viral surface, non-viral self-assembling proteins such as I50–50, ferritin, mi3, and AP205 can also be used as antigen display scaffolds (**Figure 3**) (Frietze et al., 2016; Olshefsky et al., 2022; Tursi et al., 2023). These non-viral self-assembling VLPs are being investigated with the intent of developing vaccine platforms for numerous infectious diseases (Rappuoli and Serruto, 2019; Curley and Putnam, 2022). Particularly, in recent SARS-CoV-2 vaccine development, displaying RBD antigens on VLP platforms has shown stronger immunogenicity and greater protection by mimicking the physicochemical characteristics of SARS-CoV-2 (Kang et al., 2021).

### 4.1 Potential advantages of VLP vaccines

**Figure 4:**
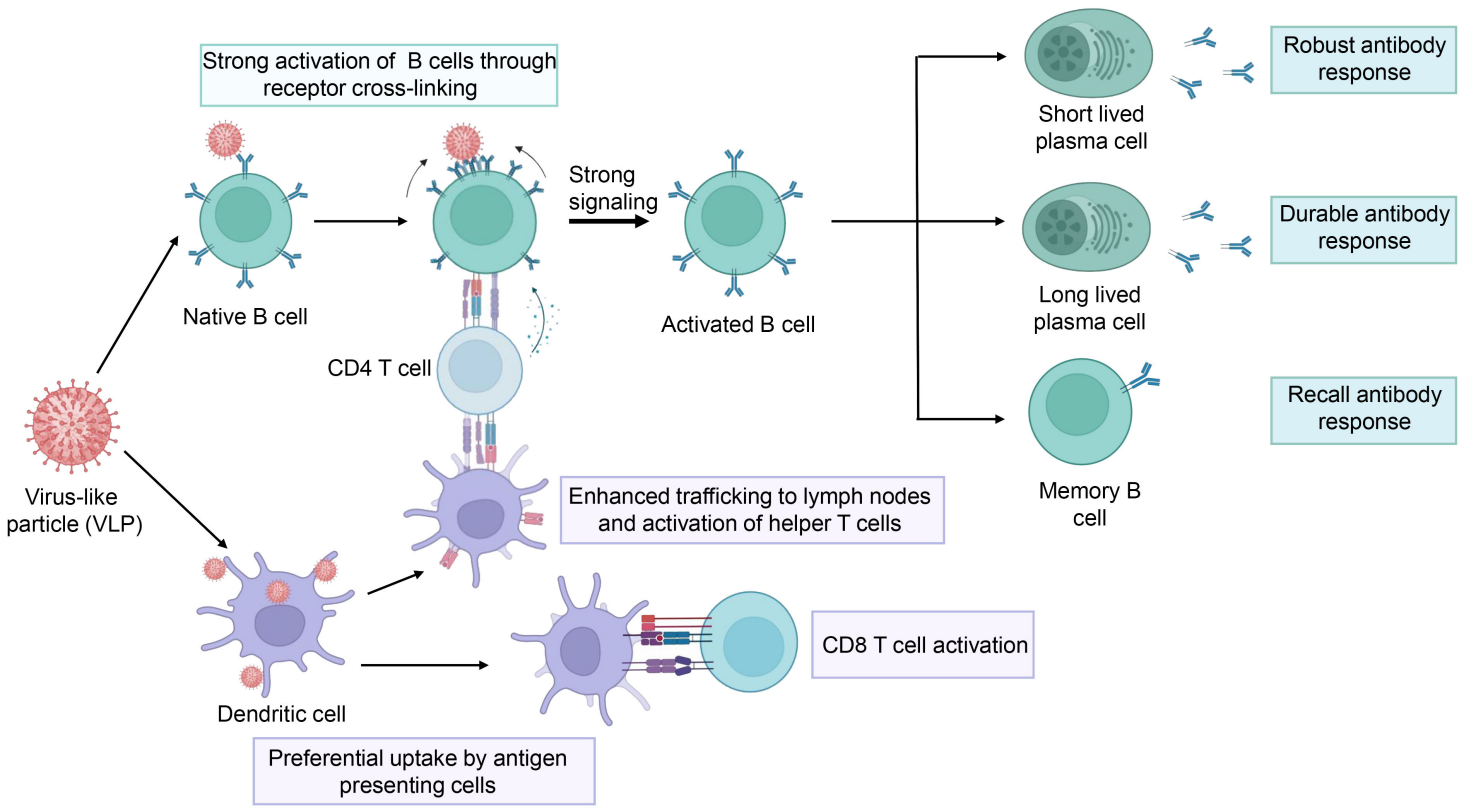
The schematic representation of the immune response elicited by the VLPs. The figure was adapted from BioRender.com.

Compared to traditional inactivated and attenuated vaccines, VLP vaccines are considered to be safer because they lack a viral genome, thereby eliminating the potential risks that stem from replication and infection. VLP vaccines typically have better immunogenicity than subunit vaccines and recombinant protein vaccines, as VLPs mimic viral size and appearance, which are highly effective in triggering the innate and adaptive immune systems (**Figure 4**) (Gomes et al., 2017). First, the size of VLPs usually ranges between 20 to 60 nm, making it easier for them to diffuse into the lymph nodes through the pores in the walls of the lymphatic vessels (Reddy et al., 2007; Manolova et al., 2008). Thus, LN-trafficking of VLPs is generally sustained longer and is more efficient than that of soluble antigens (Link et al., 2012; Mueller et al., 2015). As a result, the VLPs induce more durable antibody responses. Second, VLPs contain a precise arrangement of multiple copies of an antigen on their surface, thus modulating the resulting immunogenicity. Comparatively, soluble antigens are largely limited to engagement with a single B-cell receptor (BCR), while the multivalent antigens presented on VLPs can simultaneously engage multiple BCRs on the same cell. In addition, within lymph nodes, VLPs are not only transported to B cell follicles but are also preferentially taken up by dendritic cells and macrophages (Yue et al., 2010; Jia et al., 2012), which present processed VLP-derived antigens to CD4^+^ T cells, aiding in B cell activation. Therefore, VLPs elicit stronger B cell activation (Puffer et al., 2007; Yang and Reth, 2010). Moreover, antigens presented with particles elicited more targeted memory B cells than soluble antigens alone. Therefore, VLPs can elicit potent antibody responses (Thompson et al., 2018). Finally, VLPs can facilitate the presentation of antigens in a pathogen-like conformation for B cells to recognize, while shielding poorly neutralizing epitopes and increasing access to highly neutralizing epitopes, thus inducing broadly neutralizing antibodies (Benen et al., 2014). In addition to presenting VLP-derived antigens to CD4^+^ T cells, dendritic cells can also present VLP-derived antigens through the classical major histocompatibility complex II (MHC) pathway and MHC I molecule cross-presentation. Therefore, VLPs can trigger CD8^+^ T cell responses (Keller et al., 2010; Speiser et al., 2010; Molino et al., 2013).

However, challenges in terms of VLP-based technologies in the field of vaccines still remain. The manufacture of VLP-based vaccines requires complicated and delicate procedures to ensure the quality of the assembled particles. Most commercial vaccines use relatively simple conventional platforms. This may explain why only a few VLP-based vaccines are commercially available in the marketplace nowadays. Moreover, VLP assembly involves the assembly of single or multiple structural capsid proteins in appropriate production hosts (*in-vivo* assembly) or cell-free conditions (*in-vitro* assembly) (Kunkel et al., 2001; Roldão et al., 2012). Therefore, optimization is essential for appropriate VLP assembly. The use of transduction to separate complex protein mixtures from VLPs is also challenging, owing to their similar particle sizes and molecular weights (Vicente et al., 2011). Importantly, various techniques are required for the definitive biochemical characteristics of VLPs, and urgent improvements that can fulfill the demand for differentiating between unassembled and partially or completely assembled VLPs with higher sensitivity and effectiveness are required (Gupta et al., 2023). Thus, the high cost and process complexity of manufacturing constitute key challenges for VLP-based vaccines. Furthermore, existing commercial VLP-based vaccines are primarily used to protect against human viral infections (Hadj Hassine et al., 2024). However, the efficacy of VLP-based vaccines against other types of pathogenic microbes, such as bacteria, remains unclear.

### 4.2 Production of VLP vaccines

Currently, SARS-CoV-2-targeted VLP vaccines are primarily produced using four different particle units: I50–50, ferritin, mi3, and AP205. The I53−50 VLP was computationally designed as an icosahedral nanoparticle with 24–40 nm in diameter, co-assembled using twelve pentameric I53−50B and 20 trimeric I53−50A aligned along the fivefold and threefold symmetry axes respectively (Bale et al., 2016). The antigens were genetically fused to the N-terminus of I53–50A to assemble an intact I53–50 VLP vaccine (Marcandalli et al., 2019; Brouwer et al., 2021; Sun et al., 2023). The second particle unit, ferritin, is a spherical, self-assembling protein particle with a diameter of 12 nm, containing 24 subunits (Han et al., 2014). Isolated from *Helicobacter pylori* (*H. pylori*), ferritin has been successfully used as a VLP platform for vaccine development (Kanekiyo et al., 2015). In addition, mi3 was computationally designed based on the 2-dehydro-3-deoxy-phosphogluconate (KDPG) aldolase from *Thermotoga maritima*. In the molecular design of mi3, five substitutions (E26K, E33L, K61M, D187V, and R190A) were incorporated to generate an interface with the wild-type protein trimer, thus promoting its assembly into dodecahedral 60-mer particles with a diameter of 26 nm. Furthermore, C76A and C100A mutations were introduced to avoid potential disulfide bond-mediated heterogeneity (Hsia et al., 2016; Bruun et al., 2018). Moreover, AP205 possesses a coat protein that can self-assemble into icosahedral 180-mer particles with a diameter of 20 nm (Tissot et al., 2010; Brune et al., 2016; Shishovs et al., 2016).

All these particle units are required to artificially assemble with targeted antigens using the SpyTag-SpyCatcher covalent bond linking system. In recent years, the SpyTag-SpyCatcher covalent bond linking system has advanced significantly, making protein modification and macromolecular assembly easier, particularly for VLP vaccine development. SpyTag and SpyCatcher were obtained by splitting the *Streptococcus pyogenes*-derived fibronectin-binding protein FbaB using a rational design. SpyTag spontaneously forms an amide bond with SpyCatcher between Lys and Asn within minutes (Bruun et al., 2018). Therefore, to generate VLP vaccines, the viral antigen is displayed on the VLP scaffold using Spy-Tag/Spy-Catcher technology (He et al., 2021; Liu et al., 2021; Tan et al., 2021). In addition to the bio-conjugation technology, ferritin VLP vaccines can be developed by genetically fusing the antigen to the ferritin subunit (Kanekiyo et al., 2015; Kim et al., 2021).

### 4.3 VLP vaccines in clinical trials targeting SARS-CoV-2

To date, two SARS-COV-2 VLP vaccines have been authorized, and six vaccine candidates are currently undergoing clinical trials (**Table 2**).

**Table 2:** VLP-based SARS-COV-2 vaccine in clinical studies.

Vaccine name	Developers	Antigen	VLP carrier	Phase	NCT number
SKYCovione (GBP510)	SK bioscience + GSK	RBD of S protein	I53–50	Authorized in Korea	
COVIFENZ (CoVLP)	Medicago Inc.	Full-length S protein	Plant-derived self-assembled VLP	Authorized in Canada	
SpFN	The Walter Reed Army Institute of Research	Ectodomain of S protein	Ferritin	Phase 1	NCT04784767
COVIVAXX	Serum Institute of India + SpyBiotech	RBD of S protein	HBsAg VLP	Phase 1/2	ACTRN12620001308987
ABNCoV2	Bavarian Nordic	RBD of S protein	AP205	Phase 1 Phase 2	NCT04839146 NCT05077267
VBI-2902a	VBI Vaccines Inc.	Ectodomain of S protein	Murine leukemia virus (MLV)-based eVLPs	Phase 1	NCT04773665
LYB001	Yantai Patronus Biotech Co., Ltd.	RBD of S protein	I3–01	Phase 1 Phase 2/3 Phase 3	NCT05125926 NCT05137444 NCT05664932
IVX-411	Icosavax, Inc.	RBD of S protein	I53–50	Phase 1/2	/

#### 4.3.1 SKYCovione (GBP510)

SK Bioscience, in collaboration with GlaxoSmithKline (GSK), has developed a recombinant protein vaccine, SKYCovione (GBP510), for COVID-19. The vaccine consisted of GBP510 and an AS03 adjuvant. GBP510 is a self-assembling two-component protein virus-like particle based on the I53–50 carrier, which contains 60 copies of the receptor-binding domain (RBD) of the SARS-CoV-2 Spike protein. The RBD-I53–50A fusion protein is expressed in mammalian cells. The GBP510/AS03 vaccine induced higher neutralizing antibody titers than prefusion-stabilized spike protein in mouse and rhesus macaque models (Walls et al., 2020; Jacob-Dolan et al., 2023).

A phase 1/2 study was conducted to evaluate the safety and immunogenicity of GBP510 with two doses of 10 or 25 µg administered intramuscularly, 28 days apart, with or without AS03 in adults aged 19–85 years in Korea. Data from this early-phase trial indicated that GBP510 with an AS03 adjuvant was well-tolerated and highly immunogenic (Song et al., 2022).Furthermore, the safety and immunogenicity of the vaccine were evaluated for two doses of 25 μg of vaccine administered 28 days apart and were compared with ChAdOx1-S (Vaxzevria, AstraZeneca) in a phase 3 trial with 4036 participants aged ≥18 years in six countries (South Korea, Philippines, Thailand, Vietnam, Ukraine, and New Zealand). The results indicated that a two-dose vaccination with GBP510/AS03 induced stronger neutralizing antibody and cell-mediated immune responses than ChAdOx1-S against the ancestral D614G strain 2 weeks after the second vaccination, sustained a favorable trend to GBP510/AS03 up to 6 months, and had a clinically acceptable safety profile up to 6 months after the second vaccination (Song et al., 2023). The SKYCovione vaccine was authorized in Korea in June 2022.

#### 4.3.2 COVIFENZ (CoVLP)

COVIFENZ (Coronavirus-like particle COVID-19, CoVLP) is a recombinant plant-derived self-assembled virus-like particle vaccine developed by the Medicago Biopharmaceutical Company in Canada. The adjuvant AS03 for the COVIFENZ vaccine material was provided by GSK. The S protein of SARS-CoV-2 was expressed in *Nicotiana benthamiana* with modifications in the S1/S2 cleavage site (R667G, R668S, and R670S substitutions) to increase protein stability and stabilize the protein pre-fusion conformation by modification of K971P and V972P substitutions. The signal peptide of the S protein was replaced with a plant gene signal peptide and the transmembrane domain (TM) and cytoplasmic tail (CT) were replaced with TM/CT from Influenza H5 A/Indonesia/5/2005 to enhance VLP assembly and budding (D'Aoust et al., 2010; Dubé et al., 2022). This VLP was formulated using AS03 to develop COVIFENZ. Pre-clinical studies have shown that this vaccine effectively neutralizes SARS-CoV-2 in mice and nonhuman primates (Dubé et al., 2022; Pillet et al., 2022).

In the phase 1 clinical trial, a total of 180 participants (adults, 18–55 years) were randomized, immunized, and followed for safety and immunogenicity at two sites in Quebec, Canada, to receive two intramuscular doses of CoVLP (3.75, 7.5, and 15 μg) 21 days apart, alone or with AS03 or CpG1018 as an adjuvant. All formulations were well-tolerated. After the second dose, CoVLP/AS03 groups induced higher NAbs and spike protein-specific interferon-γ and interleukin-4 cellular responses (Ward et al., 2021). The phase 2 clinical trial demonstrated that CoVLP/AS03 is well-tolerated and highly immunogenic, generating a durable (at least 6 months) immune response against different VOCs: Alpha, Beta, Gamma, Delta, and Omicron BA.1 in adults ≥18 years of age, with and without comorbidities (Charland et al., 2022). In the phase 3, multinational, randomized, placebo-controlled trial conducted at 85 centers in Argentina, Brazil, Canada, Mexico, the United Kingdom, and the United States, a total of 24,141 adults (≥18 years of age) in a 1:1 ratio received two intramuscular injections of 3.75 μg of CoVLP/AS03 vaccine or placebo 21 days apart. The CoVLP+/AS03 vaccine was effective in preventing COVID-19 caused by a range of variants, with efficacies ranging from 69.5% against symptomatic infections to 78.8% against moderate-to-severe disease (Hager et al., 2022). COVIFENZ was approved in Canada in February 2022.

#### 4.3.3 SpFN

The Walter Reed Army Institute of Research (WRAIR) developed a SARS-CoV-2 spike ferritin VLP vaccine (SpFN) in which the spike ectodomain of SARS-COV-2 was genetically fused to the N-terminus of *Helicobacter pylori* ferritin molecule (Joyce et al., 2021a). Army Liposome Formulation containing QS-21 (ALFQ) is a powerful adjuvant that generates well-balanced TH1/TH2 immunity and protective efficacy (Carmen et al., 2021). Immunogenicity and challenge studies in mice (Joyce et al., 2021b), hamsters (Wuertz et al., 2021), and nonhuman primates (Joyce et al., 2022) following vaccination with SpFN/ALFQ showed robust antibody responses to spike proteins and protection against challenges with SARS-COV-2 and variants. A phase 1 clinical trial (NCT04784767) of SpFN+ALFQ is currently ongoing.

#### 4.3.4 COVIVAXX

COVIVAXX is a conjugated vaccine developed by SpyBiotech along with the Serum Institute of India Pvt. Co., Ltd. (SIIPL). The RBD of the SARS-CoV-2 S protein is displayed on the surface of the hepatitis B surface antigen (HBsAg) VLP based on SpyTag/SpyCatcher technology (Marini et al., 2019; Kaur et al., 2021). Pre-clinical studies have shown 100% seroconversion and high levels of antibody titers against the RBD (https://www.covidx.eu/covivaxx). Both the RBD-Spy-Tag and HBsAg SpyCatcher components are produced in yeast. A phase 1/2 trial (ACTRN12620001308987) is currently ongoing in Australia to assess the safety and immunogenicity of RB.

D-HBsAg VLP adjuvanted with Alum + CpG 1018 or alum alone, in which two doses are administered at an interval of 28 days in humans.

#### 4.3.5 ABNCoV2

The capsid-like particle (CLP)-based vaccine (ABNCoV2) was developed by Bavarian Nordics. The RBD of the SARS-CoV-2 spike protein was genetically fused to SpyCatcher, and SpyTag was genetically fused to the coat protein of AP205. RBD antigens are displayed on AP205 VLP using SpyTag/SpyCatcher technology, ensuring the unidirectional and high-density display of the RBD (Fougeroux et al., 2021). RBD-AP205 VLP formulated with Addavax adjuvant induced significantly higher levels of neutralizing antibodies and protection against challenge with SARS-COV-2 in mouse and rhesus macaque models (Fougeroux et al., 2021; Volkmann et al., 2022).

In the phase 1 clinical trial, participants were vaccinated intramuscularly on days 0 and 28 with 6, 12, 25, 50, or 70 μg of the ABNCoV2 or MF59-adjuvanted ABNCoV2. The results of this trial showed that ABNCoV2 was well tolerated and induced strong neutralizing antibody responses in healthy adults. The immune response to the vaccine antigen was dose-dependent, and MF59 showed a dose-sparing effect (Smit et al., 2023). An open label phase 2 trial (NCT05077267) is currently ongoing to evaluate the safety, tolerability, and immunogenicity of the ABNCoV2 vaccine in SARS-CoV-2 seronegative and seropositive adult subjects.

#### 4.3.6 VBI-2902a

VBI Vaccines Inc. developed a SARS-CoV-2 vaccine candidate, VBI-2902a, using murine leukemia virus (MLV)-based enveloped virus-like particles (eVLPs). VBI-2902a is a recombinant SPG-eVLP protein formulated with an alum adjuvant. The S ectodomain of SARS-CoV-2 fused with the transmembrane cytoplasmic terminal domain (TMCTD) of VSV-G was referred to as SPG, enabling the highest yield and density of S expression on MLV-Gag eVLPs. VBI-2902a was safe and highly efficacious in a Syrian golden hamster challenge model after only a single dose, supporting the on-going clinical evaluation of VBI-2902a as a highly potent vaccine against COVID-19 (Fluckiger et al., 2021). A phase 1, randomized, observer-blind, placebo-controlled study (NCT04773665) was conducted to evaluate the safety, tolerability, and immunogenicity of the one- and two-dose regimens of VBI- 2902a with 5 μg S protein content and an aluminum phosphate adjuvant or placebo injected intramuscularly.

#### 4.3.7 LYB001

Yantai Patronus Biotech designed a vaccine, LYB001, targeting the RBD of the SARS-CoV-2 Spike glycoprotein, whereby the wild-type RBD was delivered in an array on a VLP produced using split protein Tag/Catcher technology. LYB001 showed high immunogenicity affording protection against different variants in murine and NHP challenge models (Li et al., 2023b). Both phase 1 (NCT05125926) and phase 2/3 (NCT05137444) trials are intended to evaluate the safety, reactogenicity, and immunogenicity profile of LYB001 in healthy adults aged 18 years and older. LYB001 was administered intramuscularly at a three-dose regimen at 28-day intervals on days 0, 28, and 56. Furthermore, a phase 3 trial (NCT05664932) was conducted to evaluate the immunogenicity and safety of LYB001 as a booster vaccination in adults aged 18 years or older who had completed two or three inactivated COVID-19 vaccines.

#### 4.3.8 IVX-411

IVX-411 was developed by Icosavax, Inc., in which the RBD of the SARS-CoV-2 S protein is displayed on the surface of the I53–50 VLP (Walls et al., 2020). IVX-411 induces protective immunity in mice and rhesus macaques (Arunachalam et al., 2021). The ongoing phase 1/2 clinical trial aims to evaluate the safety and immunogenicity of IVX-411 in both SARS-CoV-2 naïve and previously vaccinated adults aged 18–69 years. Naïve subjects received two doses, given 28 days apart, of IVX-411 at 5, 25, or 125 µg dosage levels or placebo, with or without adjuvant. Previously vaccinated subjects were boosted with a single dose of IVX-411 at 5, 25, or 125 µg or placebo, with or without adjuvant, at 3–6 months following completion of primary licensed vaccine regimen (mRNA or adenoviral). Supplemental analysis was also conducted in this clinical trial to assess whether sera from subjects immunized with IVX-411 neutralize the SARS-CoV-2 Omicron variant.

## 5 Conclusion

Scientists are currently faced with the pressing challenge of consistently developing a new generation of precise vaccines to address the ongoing SARS-CoV-2 variant pandemic. Although inactivated, mRNA, and protein subunit vaccines are all effective, protein-based VLP vaccines remain among the safest and most effective design approaches against SARS-COV-2. Compared to the single antigen of protein subunit vaccines, protein-based VLP vaccines with an optimal size and high density of antigens present on their surface can elicit higher and more durable NAb responses. As mentioned above, VLPs can potentially serve as an effective platform for producing next-generation vaccines.

Some studies have found that different VLP carriers induce different degrees of humoral immune responses owing to differences in the diameter of the VLP carriers and the number of displayed antigens (Kanekiyo et al., 2013; Boyoglu-Barnum et al., 2021; Cohen et al., 2021b). However, no significant differences existed in inducing NAb titers against the WT and most variants of SARS-COV-2 among all the RBD-VLP vaccines (Kang et al., 2021). Therefore, the immunogenicity of novel VLP vaccines needs to be tested using different VLP carriers and antigens.

The Omicron variant has taken hold worldwide and is currently the predominant SARS-CoV-2 variant causing COVID-19, with an increasing number of infections. With the ongoing evolution of Omicron, its infectivity and immune evasion abilities continue to increase (Parsons and Acharya, 2023). In addition to the SARS-CoV-2 pandemic, two other zoonotic beta-coronaviruses, SARS-CoV and MERS-CoV, have caused regional outbreaks. An increasing number of SARS-CoVs have been identified in animals, raising concerns regarding their zoonotic transmission and the risk of future pandemics (Wong et al., 2019; Zheng et al., 2023). Some studies have indicated that immunization with a mosaic VLP vaccine (co-displaying SARS-CoV-2 RBD along with RBDs from animal beta-coronaviruses) or a multiviral quartet VLP vaccine (quartets of tandemly linked RBDs from SARS-like beta-coronaviruses coupled to the VLP carrier) could induce higher levels of neutralizing antibodies with broad-spectrum activity against SARS-like zoonotic coronavirus in animals (Cohen et al., 2021a, 2022; Hills et al., 2023). Therefore, we consider the VLP platform as a potential approach for future universal coronavirus vaccine development.

The number of patients infected with acute respiratory diseases has increased in winter. This may be related to cross-infection with various respiratory viruses, including SARS-COV-2, influenza virus, and respiratory syncytial virus (RSV) (Morens et al., 2023). Based on this, WHO issued a warning regarding co-infection with SARS-COV-2, influenza virus, and RSV. Although vaccines are available for SARS-CoV-2, RSV, and influenza viruses, no vaccine is available for all three of these viruses. Therefore, the development of multiple respiratory vaccines against these viruses is essential. To date, VLP vaccines, specifically against SARS-CoV-2, RSV, and influenza viruses have been developed based on the I53–50 VLP carrier (Marcandalli et al., 2019; Walls et al., 2020; Boyoglu-Barnum et al., 2021), indicating that it is possible to develop multiple respiratory virus vaccines utilizing the VLP platform.

## Author contributions

XY: Conceptualization, Project administration, Supervision, Writing – review & editing. XH: Conceptualization, Investigation, Methodology, Writing – original draft. FY: Data curation, Investigation, Software, Writing – original draft.
